# Serological and epidemiological investigation of *Mycobacterium avium* subspecies paratuberculosis in bovines in Pakistan

**DOI:** 10.5713/ab.23.0532

**Published:** 2024-04-25

**Authors:** Aziz ur Rehman, Muhammad Tariq Javed, Ishtiaq Ahmed, Muhammad Adnan Saeed, Syed Ehtisham-ul-Haque, Muhammad Kamran Rafique, Arbab Sikandar, Amar Nasir, Latif Ahmad, Muhammad Kashif, Muhammad Abid Zeeshan

**Affiliations:** 1 Department of Pathobiology (Pathology Section), University of Veterinary and Animal Sciences Lahore (Sub-Campus Jhang), Jhang, 35200, Pakistan; 2 Department of Pathology, Faculty of Veterinary Sciences, University of Agriculture, Faisalabad, 38000, Pakistan; 3 Department of Pathobiology (Microbiology Section), University of Veterinary and Animal Sciences Lahore (Sub-Campus Jhang), Jhang, 35200, Pakistan; 4 Department of Basic Sciences, University of Veterinary and Animal Sciences Lahore (Sub-Campus Jhang), Jhang, 35200, Pakistan; 5 Department of Clinical Sciences, University of Veterinary and Animal Sciences Lahore (Sub-Campus Jhang), Jhang, 35200, Pakistan; 6 Baqai College of Veterinary Sciences, Baqai Medical University, Karachi, 74200, Pakistan

**Keywords:** Bovine Paratuberculosis, Enzyme Linked Immunosorbent Assay (ELISA), Polymerase Chain Reaction (PCR), Tuberculin Test

## Abstract

**Objective:**

This study aimed to investigate the prevalence of paratuberculosis in cattle and buffaloes at twelve public dairy farms in Punjab, Pakistan.

**Methods:**

A total of 2,181 more than two-year-old animals (1,242 cattle and 939 buffaloes) were tested by avian tuberculin, i.e., killed purified protein derivative of *Mycobacterium avium* paratuberculosis and indirect enzyme linked immunosorbent assay (ELISA). Blood and fecal samples were collected from tuberculin positive animals. These samples were further processed by indirect ELISA. The data were analyzed using frequency analysis and logistic analysis procedures.

**Results:**

The prevalence of paratuberculosis at public dairy farms was 3.8%, as determined by tuberculin+ELISA test. It varied from 0.71% to 13.5% with a 100% herd prevalence. Multivariate logistic regression analysis revealed that species, milk production, total animals, total small ruminants, and total buffaloes were significantly associated with the occurrence of paratuberculosis. Odd ratio analysis revealed that with a one-kilogram increase in body weight, there will be a 0.006% increase in disease occurrence. With the increase in one animal in small ruminants and buffaloes, there will be 0.008% and 0.42% greater chances of developing paratuberculosis, respectively. Bivariate logistic regression analysis of cattle and buffaloes revealed that farm number, age, and total number of cattle were significantly associated with the occurrence of paratuberculosis. A one-month increase in lactation length increases the chance of tuberculosis by 0.004%; similarly, a one-liter increase in milk production increases the chance of disease by 10%. With each additional buffalo in the herd, there will be a 0.007% greater chance for the occurrence of paratuberculosis.

**Conclusion:**

This study concluded that tuberculin testing can be used in conjunction with ELISA to screen animals for paratuberculosis in countries with scarce resources, such as Pakistan. The efficacy of disease diagnosis can be improved by combining multiple tests.

## INTRODUCTION

The causative agent of bovine paratuberculosis is *Mycobacterium avium* subsp. *Paratuberculosis* (MAP). This disease mainly affects domestic and wild ruminants and results in economic losses, especially dairy animals. This disease is also of zoonotic importance because Crohn's disease patients in humans share the same organism [1]. Paratuberculosis occurs worldwide, and control programs have started in developed countries to reduce the prevalence of this infection [2]. The main reasons for controlling the disease are zoonosis and the economic losses faced by farmers. The long duration of illness and subsequent deterioration of the health of the animals have a considerable impact on productive and reproductive performance [3]. The mode of disease spread is via the fecal oral route in dairy herds.

In contrast, different routes of transmission, i.e., contaminated feed, water, and in-utero transmission, have also been reported [4]. MAP can also be found in milk and colostrum, and young calves can contract the infection. Animals infected with MAP can have persistent diarrhea, progressive weight loss, and decreased milk yield, leading to economic losses in the dairy industry [5].

Diagnosing Johne's disease at an early stage is essential for measuring disease magnitude and designing control strategies for MAP infection, especially in dairy animals. The detection of MAP is difficult due to the need for efficient diagnostic tools at the individual level. Although conventional diagnostic approaches such as tuberculin skin testing, enzyme linked immunosorbent assay (ELISA), and Ziehl Neelsen (ZN) staining could be better at determining the viability or number of organisms, these methods can also be used to identify organisms in resource limiting countries, including Pakistan. Polymerase chain reaction (PCR) is considered a comparatively rapid and precise diagnostic tool that enables early detection of disease in animals. However, PCR based disease diagnosis is often difficult because of the long-term subclinical stage, intermittent shedding of the organism in small amounts, the presence of PCR inhibitors in fecal samples, and the presence of very stable Mycobacterium cells, among other features that hinder DNA extraction to a substantial degree. A range of different diagnostic techniques, such as tuberculin testing, ZN staining, ELISA, culture isolation, and PCR are conventionally used to detect *Mycobacterium avium* subsp. *Paratuberculosis*. These tests provide the evidence needed to plan for the control strategies for MAP infection [6]. Acid-fast staining of fecal samples is less sensitive. However, serological tests such as ELISA were more sensitive for detecting paratuberculosis [7]. The combination of ELISA and fecal PCR increases the overall diagnostic sensitivity for the detection of paratuberculosis. The MAP fecal culturing method was considered the gold standard method for the diagnosis of Johne's disease, although this method is being replaced by detection of the organism by PCR [8]. The organism is present worldwide. The prevalence of herd disease has been reported to be 21.4% in Ireland [9], 19.3% in southern Chile [10] and 4% in Italy [11]. In France, an ELISA-based study revealed a 2.9% prevalence [12]. In dairy animals in the USA, the prevelance of paratuberculosis infection has been reported to reach be up to 5% to 10%, and in herds, it is 33% [13]. Another study on dairy herds in the United States reported a 9.11% prevalence [3], while in dairy herds in England, the disease prevalence was 7.3%. This study also revealed that the chances of gaining a positive test result for paratuberculosis increase with age [14]. The MAP rate in bovines is increasing, and there are significant discrepancies in routine laboratory techniques and variability in the identification of substantial risk factors [15]. As a result, strict measures are required. As only limited studies on the disease have been carried out, no study has been conducted at the farm level; therefore, there is a need for more studies to determine the status of the disease and associated risk factors. As the disease is of considerable socioeconomic importance, ensuring its present disease status in Pakistan is essential. Therefore, the present study investigated the prevalence of paratuberculosis in cattle and buffalo at selected areas/farms to evaluate various risk factors influencing disease status and compare the suitability of ELISA, purified protein derivative (PPD) and PCR for disease diagnosis.

## MATERIALS AND METHODS

### Study design

This study was conducted at twelve public livestock farms in Punjab, Pakistan, and according to the criteria, clinical disease occurs only in adult animals, so cattle and buffaloes older than two years were selected for this study. A total of 2,181 animals were screened from these public livestock farms. The animals were distributed as follows: Farm 1 (n = 403), Farm 2 (n = 140), Farm 3 (n = 106), Farm 4 (n = 169), Farm 5 (n = 165), Farm 6 (n = 178), Farm 7 (n = 271), Farm 8 (n = 149), Farm 9 (n = 184), Farm 10 (n = 135), Farm 11 (n = 80), and Farm 12 (n = 201). Various serological and epidemiological aspects of the disease paratuberculosis were studied. All animals were screened using a delayed hypersensitivity test with an intradermal injection of avian PPD on the left side of the neck and skin induration was observed after 72 hours at the injection site. The animals were categorized as positive or negative based on skin induration criteria described by Aagaard and colleagues [16]. A specialized questionnaire was designed for primary data recording, i.e., species, age, body weight, animal status, lactation number and length, and total number of animals, etc. The grouping was made according to these parameters.

### Sampling

Blood and fecal samples were collected for hematobiochemical and PCR studies.. Blood samples from positive animals were collected in both anticoagulant and plain vacutainer for hematological and serum biochemical parameters. The serum was separated and stored at −20°C for later investigation. The exact number of samples collected from the healthy animals was used as a control. Hematological indices, including the red blood cell (RBC) count, hemoglobin (Hb) concentration, mean corpuscular hemoglobin (MCH), mean cell hemoglobin concentration (MCHC), packed cell volume, white blood cell (WBC) count and differential leukocyte count were measured through a hematological analyzer (Exigo 400). Total serum proteins, albumin, and globulin were determined using commercial kits (Bioactiva, Germany) through an Optizen serum biochemistry analyzer. Serum samples were investigated utilizing an indirect ELISA for the detection of antibodies against MAP through a commercial kit (Lsivet Ruminant Serum paratuberculosis "Advanced" kit Lot No. 2-VETPTRS-007). The fecal samples were further subjected to PCR for the rapid diagnosis of paratuberculosis using specific primers and conditions with 1% agarose gel, i.e., insertion sequence (IS) 1245 F and R (GTGGGCAATCTGCCCTG CACTTCGG), (GCCCGCACGCTCACA GTTAAGCCGT) [17]. The PCR conditions were as follows: denaturation at 94°C for 2 minutes, 35 cycles of denaturation for 30 seconds, annealing temperature at 65°C for 2 minutes, and 3 elongations for 3 minutes at 72°C [18].

### Statistics

The collected data were analyzed by using frequency analysis, stratified analysis, and logistic analysis procedures, i.e., multivariate logistic and bivariate logistic regression the analysis of variance technique was also applied and the means were compared by Duncan’s multiple range test (SAS, 2007).

## RESULTS

### Prevalence of paratuberculosis

**Figure 1:**
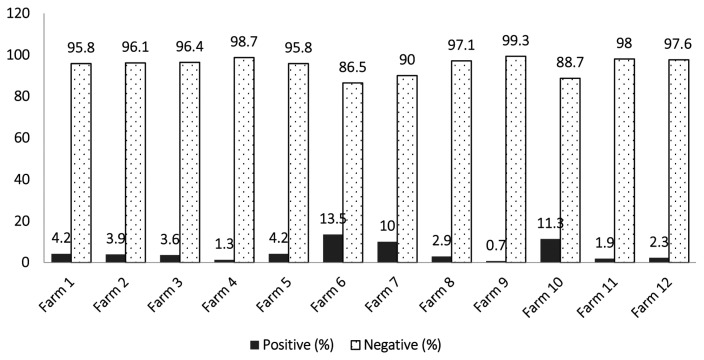
Prevalence percentage of paratuberculosis in cattle and buffaloes at twelve government livestock farms on the basis of PPD+ELISA. PPD, purified protein derivative; ELISA, enzyme linked immunosorbent assay.

**Figure 2:**
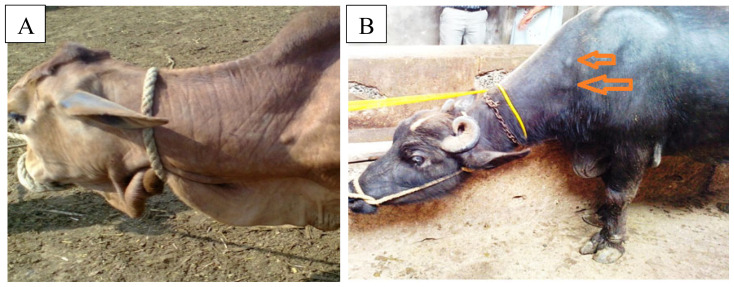
Tuberculine test positive results. The skin thickened at the ppd injection site. (A) cattle, (B) buffalo.

**Figure 3:**
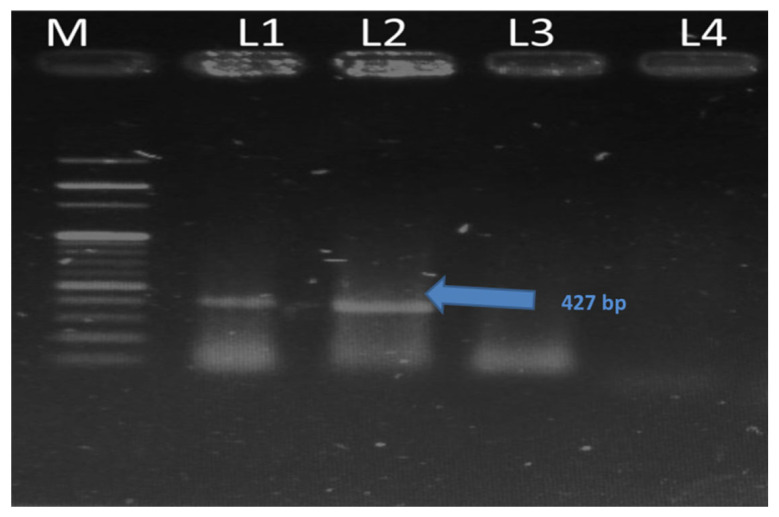
The lane 1 and 2 showed 427 bp product with primer IS 1,245, while in lane 3 and 4 no result was found. M, molecular marker.

A total of 2,181 animals, including cattle and buffaloes, were screened based on PPD+ELISA. Of these, 86 (3.8%) animals were found to be positive at government livestock experimental farms (Punjab). The disease prevalence ranged from 0.71%–13.5%. The highest prevalence (13.5%) of paratuberculosis was found at government livestock Farm-6, while the lowest prevalence (0.71%) was at livestock Farm-9 (Figure 1). The tuberculine test alone was found positive in 2.75% of animals. The skin induration of the PPD-positive animals is shown in Figure 2. PCR results were positive in 88 (4.03%) cases (Figure 3).

### Paratuberculosis in cattle and buffaloes

The prevalence ofparatuberculosis at twelve government experimental farms in Punjab, cattle and buffaloes determined by PPD+ELISA are given in Table 1. The statistical procedures, i.e., 95% confidence interval (CI) revealed a significant difference in prevalence of the two species, and the prevelance in cattle was higher (4.67%) compared to buffaloes (2.98%).

### Paratuberculosis in cattle concerning different parameters

The prevalence results of paratuberculosis in cattle of different age, weight, lactation length, milk yield, lactation number, lactation status, total animals' density, and number of small ruminant groups at twelve public dairy farms according to tuberculin (PPD)+ELISA are given in Table 2. The statistical analysis, chi-square test, and 95% CI revealed nosignificant differences in prevalence between the age, weight, lactation length, and milk production groups. The prevalence was relatively higher in animals aged between 5.1 to 8 years and heavy animals. The statistical procedures, i.e., chi-square analysis and 95% confidence interval, verified a significant (p<0.05) difference in prevalence between the four lactation length groups and the four milk yield groups. A high rate of disease was present in animals with lactation lengths of >6 months and in high-milk-producing animals. Taking lactation number and cattle stock into account, the lactation status of the animals revealed a statistically non-significant difference in disease prevalence among these groups. A somewhat higher prevalence rate was detected in lactating animals. According to the total stock density of small ruminants present at the dairy farm, the 95% confidence limits revealed a significant difference, with a lower prevalence in cattle when the total number of small ruminants was between 500–1,000. Multivariate logistic regression analysis with a backward elimination procedure in cattle showed that farm number, weight, total animals, total small ruminants, total cattle, and total buffaloes were positively associated with the occurrence of paratuberculosis (Tables 3, 4). With a one-kilogram increase in body weight there will be a 0.006% more chance of disease occurrence, and with an increase of one animal in the total number of small ruminants and the total number of buffaloes, there will be 0.008% and 0.42% more chances of the occurrence of paratuberculosis, respectively. Bivariate logistic regression analysis of cattle showed that farm number, lactation length, milk production, total animals, and total buffaloes showed significant association with the occurrence of paratuberculosis (Table 3). A one-month increase in lactation length increases the chance of tuberculosis by 0.004%; similarly, a one-liter increase in milk production increases the chance of disease by 10%. With each additional buffalo in the herd, there will be a 0.007% more chance for the occurrence of paratuberculosis. After controlling the age as a constant factor, the bivariate logistic analysis showed that farm number, lactation length, milk production, and total buffaloes were significantly associated with the occurrence of paratuberculosis (Tables 5, 6).

### Comparison between PPD and ELISA, considering PCR as the gold standard

The PPD and ELISA resultsfor the PCR positive+negative cases are presented in Table 7. Considering PCR as the gold standard, the PPD sensitivity and specificity were 68.2% and 98.9%, respectively, while for ELISA, the sensitivity and specificity were 97.7% and 100%, respectively.

### Hematobiochemical studies

Blood with and without anticoagulant was collected from infected and control animals. Anticoagulant containing blood samples were used for hematological studies (Table 8). The present study showed a significant difference between the two control groups (positive, negative) in terms of most parameters, such as RBCs, MCHC, and MCH, while the platelet cound decreased significantly in the positive reactor animals. At the same time, WBCs, lymphocytes, and granulocytes were significantly higher in positive animals. Without anticoagulants, blood samples were used for serum separation. This blood was further used to determine serum biochemical changes (Table 9). The results revealed that the total protein, albumin, and globulin levels in the infected animals were significantly lower than those in the non-infected animals.

## DISCUSSION

Paratuberculosis, also called Johne's disease, is caused by *Mycobacterium avium* subsp. *paratuberculosis* [19]. MAP disrupts either the fusion of phagosomes with lysosomes or inhibits the generation of oxygen radicals necessary for bacterial elimination [20]. Most of the time, the organism is thought to go through the small intestine's lumen and move toward Peyer's patches [21]. From a zoonotic aspect, the disease is of imperative importance, and MAP is assumed to have a probable role in the cause of Crohn's disease in humans [22]. The current study investigated the incidence of paratuberculosis in cattle and buffalo in different districts of Punjab Province. In Pakistan, different studies on the prevalence of tuberculosis have been done at different times and on various animal species. The current study found 3.8% paratuberculosis prevalence using PPD and ELISA. In Contrast, PCR Confirmed 4.03% cases.

This has led to a variable prevalence. Similar findings were observed in a study planned at two abattoirs in Jhang for cattle and buffaloes. This research confirmed a prevalence of 11.19%, according to ELISA [23].

The results from these two species showed that the disease was more common in cattle (4.67%) than in buffaloes (2.98%). The prevalence of paratuberculosis was 5.56% and 5.88% in animals (buffaloes and cattle, respectively), which indicated that the prevalence was higher in cattle [24]. Similarly, a study revealed that the prevalence of paratuberculosis was 0.5% in buffaloes and 1.5% in cattle [25]. In Italy, the disease prevalence in dairy animals appears to be 2.8% to 5.5% [26]. In Iran, disease prevalence was as high as 12% [27]. The prevalence in India was 15.14% to 18.33% [28]. In buffaloes, data analysis revealed a significant (p<0.05) difference in prevalence between the three age groups, which was higher in older animals. The data analysis revealed a non-significant difference (p>0.05) in prevalence between the three weight groups. However, a comparatively higher prevalence was observed in heavy animals. Higher prevalence was found in the group having a lactation length of >6 months. Buffaloes had no significant difference (p>0.05) across the four lactation length groups. The group with >6 months of lactation exhibited a higher prevalence ratio. An exploratory study revealed that the prevalence of paratuberculosis was comparatively higher in animals at longerlactation stages [29]. The probable reason was that the concentration of the antibodies varied across lactation, and concentration was high in the advanced stages of lactation. In cattle, there was a significant difference (p<0.05) in the prevalence of paratuberculosis among the four milk yield groups. The disease incidence rate was higher in more productive animals. The results showed a non-significant difference between the four milk yield groups in buffaloes. Research on seroprevalence in Irish dairy herds showed that the disease was mostly present in high-producing animals [30]. According to these results, it can be suggested that the prevalence rate of paratuberculosis which is relatively high in high-yielding animals, can cause stress and thus make such animals prone to paratuberculosis.

According to lactation number, the cattle and buffaloes were divided into five groups, i.e., 0, 1–3, 3–6, 7–10, and >10. In cattle and buffaloes, a non-significant difference in the prevalence of paratuberculosis among five lactation number groups was observed. However, the group with lactation numbers 3–6 had a comparatively higher prevalence. In Denmark, a study on paratuberculosis regarding lactation number indicated that disease prevalence was higher in high lactation number groups [28]. Speculative research revealed that second and third lactation animals are more susceptible to disease, and ELISA produced more antibodies in the higher parity stage. According to status, two cattle and buffalo groups were made, i.e., dry and lactating.

In cattle and buffaloes, the results showed a non-significant difference in prevalence between dry and lactating animals; however, the disease was more prevalent in lactating animals. According to the total number of buffaloes, three groups were made, i.e., 0, 1–800, and >800. The statistical analysis revealed a statistically significant difference in prevalence (p<0.05)among the three groups. The prevalence was higher, the number of animals was above 800. In the case of small ruminants on buffalo farms, the study's findings were more favorable on farms with a higher total number of small ruminants [20]. A survey organized in New Zealand on the epidemiological aspects of disease in sheep and cattle showed a positive relationship between grazing of one animal species and that of others (co-grazing). This higher prevalence rate might be due to the grazing of different species on the same form of pasture [21]. According to the animals' total density present at farms, the results revealed that the prevalence was higher at those farms where the animals' density was high. A survey of Michigan dairy cows was conducted to determine herd prevalence. This study revealed that herds with more than 200 animals were more prone to Johne's disease, and constant infection was present at dairy farms with high stock density. Overall, multivariate logistic regression analysis with a backward elimination procedure in cattle and buffaloes at twelve livestock farms revealed that specie, milk production, total animals, total small ruminants, and total buffaloes were significantly associated with the occurrence of paratuberculosis while, bivariate logistic regression analysis revealed that farm number, age, and total number of cattle were significantly associated with the occurrence of paratuberculosis. After controlling age as a constant factor, the bivariate logistic analysis revealed that farm number, lactation length, milk production, and total buffaloes were significantly associated with paratuberculosis.

The present study included tuberculin testing; different researchers have worked on tuberculin screening, such as in a study carried out by Lilenbaum [31], which revealed that tuberculin test is a reasonable diagnostic tool, but it may interfere with reactions produced by other mycobacteria so cross-reactivity issues may occur [32,33]. Similarly, Varges et al [34] reported that intradermal tuberculin interferes with the animals' immune system and can cross-react with other mycobacteria. In tuberculin testing, this cross reaction can cause false-positive results and interfere with ELISA results. ELISA and PCR tools were also used to further investigate and validate investigate further this PPD. Most of researchers in the field of paratuberculosis have applied this diagnostic approach [35]. It can be concluded from the present study that tuberculin testing (PPD) can be used in conjunction with ELISA test for screening animals for paratuberculosis in resource-poor countries. The study concluded that PPD, ELISA, and PCR are efficient diagnostic tools to diagnose the paratuberculosis, and the combination of these tests can improve the efficiency and confirmation of the disease.

## Figures and Tables

**Table 1:** The association of paratuberculosis with species using PPD+ELISA

Specie	Tuberculin+ELISA		

	Negative	Positive (%)	95% CI
Cattle	1,184	58 (4.67)	3.60–4.95
Buffalo	911	28 (2.98)	2.03–4.22
	M-H Chi-square p = 0.04		

PPD, purified protein derivative; ELISA, enzyme linked immunosorbent assay; CI, confidence interval.

**Table 2:** Paratuberculosis prevalence in cattle and buffaloes as measured by PPD+ELISA at twelve public dairy farms

Parameters	Cattle			Buffaloes		
	
	Negative	Positive (%)	95% CI	Negative	Positive (%)	95% CI
Age (yr)						
1–5	218	11 (4.80)	2.55–8.19	106	1 (0.93)	0.05–4.52
6–8	417	26 (5.87)	4.05–8.44	398	8 (1.97)	0.92–3.71
>8	549	21 (3.68)	2.36–5.48	407	19 (4.46)	2.79–6.75
	M-H chi-square p = 0.29			M-H chi-square p = 0.01		
Weight (kg)						
<300	239	7 (2.85)	1.25–5.55	-	-	-
300–500	931	49 (5)	3.76–6.50	419	13 (3.01)	1.68–4.96
>500	14	2(12.50)	2.15–35.5	492	15 (2.96)	1.73–4.73
	M-H chi-square p = 0.06			M-H chi-square p = 0.96		
Lactation length						
0	98	1 (1.01)	0.05–4.88	68	1 (1.45)	0.07–6.94
1–3	24	1 (4.0)	0.20–18.19	2	0 (0.00)	0.00–77.6
3.1–6	163	5 (2.98)	1.10–6.47	46	1 (2.13)	0.11–10.05
>6	899	51 (5.37)	4.07–6.94	795	26 (3.17)	2.12–4.54
	M-H Chi-square p = 0.03			M-H chi-square p = 0.37		
Milk yield (L)						
0	98	1 (1.01)	0.05–4.88	68	1 (1.45)	0.7–6.94
1–4	137	7 (5.1)	2.2–9.5	47	1 (2.08)	0.10–9.85
4.1–8	702	33 (4.49)	3.16–6.18	748	25 (3.23)	2.15–4.67
>8	247	17 (6.44)	3.92–9.91	48	1 (2.04)	0–9.65
	M-H chi-square p = 0.05			M-H chi-square p = 0.51		
Lactation number						
0	23	1 (4.17)	0.21–18.8	5	0 (0)	0.00–45.07
1–3	569	26 (4.37)	2.94–6.25	437	15 (3.32)	1.94–5.29
3–6	367	20 (5.17)	3.27–7.73	225	10 (4.26)	2.18–7.45
7–10	184	10 (5.15)	2.65–8.99	200	3 (1.48)	0.38–3.97
>10	41	1 (2.38)	0.12–11.19	44	0 (0)	00–6.58
	M-H Chi-square p = 0.87			M-H chi-square p = 0.15		
Milking status						
Dry	508	24 (4.51)	2.98–6.54	423	12 (2.76)	1.50–4.64
Lactating	676	34 (4.79)	3.39–6.44	488	16 (3.17)	1.89–5.00
	M-H Chi-square p = 0.81			M-H chi-square p = 0.70		
Total cattle						
<400	501	20 (3.84)	2.43–5.76	-	-	-
400–800	258	17 (6.18)	3.76–9.52	-	-	-
>800	425	21 (4.71)	3.02–6.99	-	-	-
	M-H Chi-square p = 0.48					
Total buffaloes						
0	-	-	-	624	17 (2.65)	
1–800	-	-	-	258	3 (1.15)	
>800	-	-	-	29	8 (21.62)	
				M-H chi-square p = 0.003		
Total small ruminants						
0	348	24 (6.45)	4.27–9.31	284	4 (1.39)	0.44–3.32
500–1,000	541	11 (1.99)	1.05–3.44	425	15 (3.41)	1.99–5.44
>1,000	295	23 (7.23)	4.75–10.49	202	9 (4.27)	2.10–7.68
	M-H Chi-square p = 0.70			M-H chi-square p = 0.06		

PPD, purified protein derivative; ELISA, enzyme linked immunosorbent assay; CI, confidence interval.

**Table 3:** Multivariate and bivariate logistic regression with backward elimination procedures showed significant association in cattle

Parameter	Odd ratio	95% confidence limit	p-value
Multivariate logistic regression			
Farm number	0.505	0.330–0.774	0.0017
Weight (kg)	1.006	1.000–1.011	0.0401
Total animals	0.992	0.986–0.999	0.0168
Total small ruminants	1.008	1.001–1.014	0.0233
Total cattle	0.995	0.992–0.998	0.0007
Total buffaloes	1.042	1.016–1.068	0.0013
Bivariate logistic regression			
Farm number	0.905	0.829–0.987	0.0247
Lactation length (mo)	1.004	1.000–1.007	0.0278
Milk production (L)	1.100	0.999–1.210	0.0520
Total animals	1.000	1.000–1.001	0.0064
Total buffaloes	1.007	1.005–1.009	<0.0001

**Table 4:** Multivariate and bivariate logistic regression with backward elimination procedures showed significant association in buffaloes

Parameter	Odd ratio	95% confidence limit	p-value
Multivariate logistic regression			
Total cattle	0.995	1.002–1.005	<0.0001
Bivariate logistic regression			
Farm number	0.905	0.829–0.987	0.0247
Lactation length (mo)	1.004	1.000–1.007	0.0278
Milk production (L)	1.100	0.999–1.210	0.0520
Total animals	1.000	1.000–1.001	0.0064
Total buffaloes	1.007	1.005–1.009	<0.0001

**Table 5:** After controlling age bivariate logistic regression for parameters showing significant association in cattle

Parameter	Odd ratio	95% confidence limit	p-value
Farms number	0.905	0.829–0.987	0.0247
Lactation length (mo)	1.004	1.000–1.008	0.0119
Milk production	1.111	1.012–1.221	0.0276
Total animals	1.000	1.000–1.001	0.0053
Total buffaloes	1.007	1.005–1.009	<0.0001

**Table 6:** After controlling the age bivariate logistic regression procedures in buffaloes

Parameter	Odd ratio	95% confidence limit	p-value
Total cattle	1.002	1.001–1.003	<0.0001
Total buffalo	1.002	0.998–1.006	0.246185

**Table 7:** Relationship between PCR, ELISA, and PPD at Twelve Dairy farms

ELISA	PCR positive		PCR negative	
	
	PPD positive	PPD negative	PPD positive	PPD negative
Positive	60	26	0	0
Negative	0	2	23	2,070
	PPD sensitivity = 68.2%		ELISA test sensitivity = 97.7%	
	PPD specificity = 98.9%		ELISA test specificity =100%	
	False +Ve =1.1%		False +Ve = 0.0%	
	Both tests sensitivity = 68.2%		Both tests specificity = 100%	
	ELISA revealed more sensitive while PPD is more specific			

PCR, polymerase chain reaction; PPD, purified protein derivative; ELISA, enzyme linked immunosorbent assay.

**Table 8:** Comparison of hematological parameters between PPD positive and negative reactor cattle and buffaloes

Parameters	Positive Mean±SD	Negative Mean±SD
RBC (10^6^/μL)	3.79±0.082^A^	3.66±0.199^B^
PCV (%)	15.59±2.73	14.89±1.02
HGB (g/dL)	6.72±0.194^A^	6.22±0.95^B^
MCHC	41.85±2.25^A^	37.13±5.82^B^
MCH	23.94±0.60^A^	23.35±0.57^B^
WBC (10^3^/μL)	7.56±2.40^B^	13.73±0.89^A^
LYMPH	4.11±0.49^B^	5.032±0.26^A^
LYMPH (%)	45.86±1.60^B^	74.96±3.43^A^
GRAN	3.37±0.09^B^	3.70±0.73^A^
GRAN (%)	39.30±0.80^B^	42.40±3.79^A^
PLT	324.0±153.92^A^	690.12±159.10^B^

SD, standard deviation; RBC, red blood cells; PCV, pack cell volume; HGB, heamoglobin; MCHC, mean corpuscular heamoglobin concentration; MCH, mean corpuscular heamoglobin; WBC, white blood cells; GRAN, granulocytes; PLT, platelets.

A,B The values with different superscripts are significantly different between the groups.

**Table 9:** Comparison of serum protein alterations in PPD positive and negative reactor cattle and buffaloes

Parameters	Negative mean±SD	Positive mean±SD
Total protein	7.79±0.29^A^	6.36±0.363^B^
Albumin	3.85±0.44^A^	3.26±0.489^B^
Globulin	3.60±0.183^A^	3.22±0.385^B^

SD, standard deviation.

A,B The values with different superscripts are significantly different between the groups.
